# Resurgence of cucurbit downy mildew in the United States: Insights from comparative genomic analysis of *Pseudoperonospora cubensis*

**DOI:** 10.1002/ece3.3194

**Published:** 2017-07-03

**Authors:** Anna Thomas, Ignazio Carbone, Kisurb Choe, Lina M. Quesada‐Ocampo, Peter S. Ojiambo

**Affiliations:** ^1^ Center for Integrated Fungal Research Department of Entomology and Plant Pathology North Carolina State University Raleigh NC USA; ^2^ Department of Entomology and Plant Pathology North Carolina State University Raleigh NC USA

**Keywords:** coalescent analysis, cucurbits, disease resurgence, downy mildew, lineage, phylogenetic analysis

## Abstract

*Pseudoperonospora cubensis*, the causal agent of cucurbit downy mildew (CDM), is known to exhibit host specialization. The virulence of different isolates of the pathogen can be classified into pathotypes based on their compatibility with a differential set composed of specific cucurbit host types. However, the genetic basis of host specialization within *P. cubensis* is not yet known. Total genomic DNA extracted from nine isolates of *P. cubensis* collected from 2008 to 2013 from diverse cucurbit host types (*Cucumis sativus, C. melo* var. *reticulatus, Cucurbita maxima*,* C. moschata*,* C. pepo,* and *Citrullus lanatus*) in the United States were subjected to whole‐genome sequencing. Comparative analysis of these nine genomes confirmed the presence of two distinct evolutionary lineages (lineages I and II) of *P. cubensis*. Many fixed polymorphisms separated lineage I comprising isolates from *Cucurbita pepo*,* C. moschata,* and *Citrullus lanatus* from lineage II comprising isolates from *Cucumis* spp. and *Cucurbita maxima*. Phenotypic characterization showed that lineage II isolates were of the A1 mating type and belonged to pathotypes 1 and 3 that were not known to be present in the United States prior to the resurgence of CDM in 2004. The association of lineage II isolates with the new pathotypes and a lack of genetic diversity among these isolates suggest that lineage II of *P. cubensis* is associated with the resurgence of CDM on cucumber in the United States.

## 1 INTRODUCTION

*Pseudoperonospora cubensis* is an obligate biotrophic pathogen and is the causal agent of downy mildew of cucurbits. Cucurbit downy mildew (CDM) is considered the most economically important disease of cucurbits worldwide. The disease is distributed widely in temperate and semiarid regions, and the pathogen infects several members of the family Cucurbitaceae that includes wild and cultivated cucurbits (Lebeda, 1992). Approximately 60 species of cucurbits have been identified as hosts of *P. cubensis,* and the most economically important crop plants include cucumber, cantaloupe, watermelon, pumpkin, and squashes (Lebeda, 1992). In the United States, downy mildew was primarily a problem only on squashes, watermelon, and pumpkin prior to 2004 and the disease on these cucurbits was effectively managed using fungicides. Successful breeding efforts in the 1960s led to availability and wide deployment of resistant cucumber cultivars, and this rendered CDM on cucumber to be of minor concern requiring very limited application of fungicides to control the disease. However, in 2004, CDM resurged on cucumber, and *P. cubensis* overcame host resistance due to the *dm‐1* gene that had been effective for more than 40 years (Criswell, Call, & Wehner, 2010). Severe disease epidemics were reported in the eastern United States in 2004 and 2005, leading to complete crop failure in many fields (Holmes, Wehner, & Thornton, 2006). The disease now occurs annually in the United States, and fungicides need to be applied during the season to effectively control the disease on cucumber.

Considerable efforts have been undertaken to establish possible causes of the resurgence of CDM in the United States (Colucci, 2008; Holmes, Ojiambo, Hausbeck, Quesada‐Ocampo, & Keinath, 2015; Ojiambo, Gent, Quesada‐Ocampo, Hausbeck, & Holmes, 2015; Quesada‐Ocampo et al., 2012), in Israel (Cohen et al., 2015), and in parts of Europe (Kitner et al., 2015; Lebeda, Pavelková, Sedláková, & Urban, 2013). It has been hypothesized that the resurgence of CDM in the United States was due to the introduction of a new pathotype or a new cryptic species of the pathogen (Colucci, 2008; Runge, Choi, & Thines, 2011). A new genetic recombinant from sexual reproduction has also been suspected as a possible cause for the resurgence in other parts of the world (Cohen & Rubin, 2012; Runge et al., 2011). During the past two decades, changes in the virulence structure of *P. cubensis* have been reported around the globe. For example, new pathotypes have been reported in several countries (Cohen et al., 2015) and a change in population dynamics of the pathogen has been reported in the Czech Republic (Kitner et al., 2015; Lebeda et al., 2013). In addition, both mating types of *P. cubensis* have been found in Israel, China, the United States, and several other countries (Cohen & Rubin, 2012; Cohen et al., 2013, 2014, 2015; Thomas, Carbone, Cohen, & Ojiambo, 2017). The global resurgence of CDM has been reviewed (e.g., Cohen et al., 2015; Holmes et al., 2015; Ojiambo et al., 2015), and understanding the genetic basis for host specialization within *P. cubensis* was identified as an important component in providing insights in the resurgence of CDM around the globe (Cohen et al., 2015).

Unlike most other downy mildew pathogens, *P. cubensis* is able to infect a wide range of cucurbits (Runge et al., 2011), and the virulence of the pathogen has been classified into pathotypes based on its differential compatibility with different cucurbit hosts. A study examining the physiological specialization of *P. cubensis* using isolates from Japan, Israel, and the United States conducted in 1987 identified the existence of five different pathotypes (Thomas, Inaba, & Cohen, 1987) using thirteen differential host types namely *Cucumis sativus, C. melo* var. *reticulatus, C. melo* var. *conomon, C. melo* var. *acidulus, Citrullus lanatus, Cucurbita maxima*,* C. pepo*,* C. moschata, Benincasa hispida, Luffa acutangula*,* L. cylindrica*,* Momordica charantia, and Lagenaria siceraria*. Isolates capable of infecting *Cucumis sativus* and *C. melo* var. *reticulatus* were classified as pathotype 1, while those capable of infecting *C. melo* var. *conomon* in addition to the former hosts were classified as pathotype 2. Isolates capable of infecting all *Cucumis* spp. were classified as pathotype 3, while all isolates capable of infecting *C. lanatus* in addition to the *Cucumis* spp. were designated as pathotype 4. All isolates capable of infecting *Cucurbita* spp. in addition to *Cucumis* spp. and *C. lanatus* were classified as pathotype 5. Since then, up to 10 different pathotypes have been identified (Cohen et al., 2015). While these studies indicate a unique level of interaction between *P. cubensis* and cucurbit host types, the genetic basis underlying host specialization within this pathosystem is still unresolved.

Following the resurgence of CDM in 2004, a change in the virulence pattern of *P. cubensis* exhibited by increased aggressiveness on cucumber and a broader host range was reported in the United States (Colucci & Holmes, 2007; Thomas, Carbone, Lebeda, & Ojiambo, 2017). A phylogenetic study conducted in Europe showed that *P. cubensis* is composed of two cryptic species (Runge et al., 2011). Clade 1 (cryptic species 1) was associated with isolates in the United States collected prior to the CDM resurgence, and clade 2 (cryptic species 2) was associated with isolates from East Asia and Europe and also included one isolate from the United States collected after the 2004 resurgence. Based on the association of the post‐resurgence isolate from the United States with clade 2, it was hypothesized that the resurgence of CDM in the United States could have been due to migration of new strains, possibly from East Asia (Runge et al., 2011). However, only one isolate from the United States collected after the CDM resurgence was included in the study by Runge et al. (2011), and this lack of data precludes gaining key insights into population‐level processes that may have been initiated and subsequently progressed after the resurgence of CDM in 2004. More recent studies have documented the existence of A1 and A2 mating types of *P. cubensis* in different parts of the world (Cohen et al., 2015) including the United States (Thomas, Carbone, Cohen, et al., 2017). In the Czech Republic, the A1 mating type that has an affinity for *Cucumis* species was associated with CDM epidemics before 2009, while the A2 mating type that frequently occurs on other cucurbit host types has been associated with disease epidemics since 2009 (Lebeda et al., 2014). Lebeda et al. (2014) provided some indirect insights into host specialization within *P. cubensis* and the possible association of cucurbit host types with specific clades of the pathogen.

The evolutionary origin of *P. cubensis* and the relatedness to its sister species, *P. humuli* that infects hop, have been widely investigated (Choi, Hong, & Shin, 2005; Göker, Voglmayr, Riethmüller, & Oberwinkler, 2007; Kitner et al., 2015; Mitchell, Ocamb, Grünwald, Mancino, & Gent, 2011; Runge & Thines, 2012; Runge et al., 2011). Choi et al. (2005) conducted a phylogenetic analysis of *P. cubensis* and *P. humuli* using the nuclear ribosomal internal transcribed spacer (nrITS) region and showed a high level of sequence similarity between the two species. Based on this observation and the high degree of morphological similarity between the two species, *P. humuli* was reduced to a taxonomic synonym of *P. cubensis*. However, a subsequent study by Sarris et al. (2009) based on amplified fragment length polymorphism fingerprinting and ITS sequence variation in *P. humuli* and *P. cubensis* from *C. sativus* in Europe reported that the two species formed two distinct clusters. Mitchell et al. (2011) examined variation in three nuclear and two mitochondrial loci and reported that *P. humuli* from Europe and the United States formed a distinct clade compared to *P. cubensis* isolated from herbarium samples from South Korea. In that study (Mitchell et al., 2011), one *P. humuli* isolate from Korea clustered with *P. cubensis* isolates from Asia, whereas *P. humuli* isolates from Europe and the United States formed a separate cluster ancestral to *P. cubensis*. Some cohesion between the two species was observed in the *cox*2 region of two isolates obtained from *Cucurbita pepo* in North Carolina and a *P. humuli* isolate from South Korea (Mitchell et al., 2011), and between *P. humuli* and *P. cubensis* isolates from non‐cucumber host types (Kitner et al., 2015). Generally, these studies indicate that although *P. cubensis* and *P. humuli* are morphologically and physiologically similar, they exhibit quantifiable physiological and genetic differences that support their retention as two separate species. However, a more extensive population genetic analysis is needed to determine whether these two species are specialized on their respective hosts.

The overall goal of our study was to examine patterns of genomewide variation underlying host specialization in *P. cubensis* and to provide insights into the evolutionary processes that may have driven the resurgence of CDM with increased virulence on cucumber in the United States in 2004. To achieve this goal, this study focused on two specific objectives: (1) to examine genomewide variation in *P. cubensis* and determine whether it is associated with host specialization, and (2) to determine whether distinct evolutionary lineages or individual multilocus genotypes are associated with pathotypes and mating types of *P. cubensis*.

## 2 MATERIALS AND METHODS

### 2.1 Reference isolates

Nine single‐lesion isolates of *P. cubensis* obtained from cucumber, cantaloupe, pumpkin, butternut squash, acorn squash, and watermelon from different regions of the United States from 2008 to 2013 were used as reference isolates in this study (Table 1). These isolates were maintained in the laboratory by propagating them on their respective host types using a detached leaf assay. Briefly, adaxial sides of detached leaves of 4‐week‐old plants were placed on moist paper towels in clear acrylic boxes, and the abaxial side of the leaf was inoculated with sporangial suspension at a concentration of 2 × 10^4^ sporangia/ml. Inoculated leaves were then incubated in a growth chamber at 21/18°C under 12‐hr/12‐hr light/dark cycle and were re‐inoculated once sporangia were produced in abundance. The assay was repeated as necessary until the isolates were ready for use in the subsequent experiments.

**Table 1:** Source, description, and characterization of reference isolates of *Pseudoperonospora cubensis* collected across the United States to study the genetic basis of host specialization

Isolate ID	Original host	Year	State	County	Mating type	Pathotype
A11	Cucumber	2012	NC	Johnston	A1	3
08A1	Cucumber	2008	CA	Salinas	A1	3
2013B17	Cantaloupe	2013	NY	Ontario	A1	1
2013C3	Giant pumpkin	2013	NC	Johnston	A1	1
D3	Butternut squash	2012	SC	Charleston	A2	5
2013D6	Butternut squash	2013	AL	Escambia	A2	5
2013E1	Watermelon	2013	FL	Collier	A2	4
08F1	Acorn squash	2008	GA	Tift	A2	6
2013F2	Acorn squash	2013	SC	Charleston	A2	5
Ph^a^	Hop	2013	OR	Marion	–	–

a Refers to *Pseudoperonospora humuli* isolate OR502AA described in Withers et al. (2016).

### 2.2 Mating type determination

The mating type of the reference isolates was determined by co‐inoculating each isolate with a known A1 and/or A2 tester strain as described by Cohen and Rubin (2012). The A1 tester strain was originally isolated from cucumber in North Carolina in 2012, while the A2 tester strain was originally isolated from butternut squash in Israel. Equal volumes (1:1) of sporangial suspension (2 × 10^4^ spores/ml) of the tester strain and each isolate were mixed together, and the mixed suspension was then used to inoculate cantaloupe or cucumber leaves. Cantaloupe and cucumber are a favorable host substrate that supports maximum production of oospores (Cohen & Rubin, 2012). The first leaves of 3‐week‐old cucumber and cantaloupe leaves maintained in the glasshouse were used for inoculations. The adaxial side of detached leaves was placed on moist paper towels in clear acrylic boxes, and the abaxial side was spot‐inoculated on at least 20 different spots with 10 μl of the sporangial mixture. Care was taken to dispense the inoculum suspension at vein junctions especially for cucumber to ensure maximum infection (Thomas, Carbone, Cohen, et al., 2017). Inoculated leaves were incubated in a growth chamber at 21°C at 50 to 60% relative humidity under 12‐hr/12‐hr light/dark cycle. At 7 to 10 days postinoculation, leaf disks measuring 11 mm diameter were randomly cut from infected leaves and clarified for 24 hr in ethyl alcohol–acetic acid solution (3:1 v/v). Clarified leaf disks were washed three times in de‐ionized water and examined for the presence of oospores using a compound microscope (×100 magnification). If oospores were produced when the unknown isolate was co‐inoculated with an A1 or A2 tester strain, then that isolate was considered to be of the A2 or A1 mating type, respectively.

### 2.3 Pathotype determination and designation

An internationally recognized host differential set (Lebeda & Widrlechner, 2003) composed of *Cucumis sativus*,* C. melo reticulatus*,* C. melo acidulus*,* C. melo conomon*,* Citrullus lanatus*,* Cucurbita moschata*,* C. pepo*,* Luffa cylindrica*,* Lagenaria siceraria*,* Benincasa hispida,* and *Momordica charantia*, was used to determine the pathotype of the isolates. Poinsett 76, a cucumber cultivar with *dm‐1* gene, was also included as control to determine isolates that were capable of causing disease on previously resistant cucumber cultivars. The compatibility of the isolates with the host differential set was determined using a detached leaf assay. First and second true leaves from four‐week‐old host differentials placed with the adaxial side on moist paper towels in clear acrylic boxes were spray‐inoculated with sporangial suspension at a concentration of 5 × 10^3^ sporangia/ml. Inoculated leaves were then incubated in a growth chamber at 21/18°C under 12‐hr/12‐hr light/dark cycle. Disease severity was recorded on a per‐leaf basis by assessing the percentage of leaf area infected 5 to 7 days after inoculation (Thomas, Carbone, Cohen, et al., 2017). Two leaf disks measuring 9 mm in diameter were cut from each leaf at 7 DPI and shaken well in microcentrifuge tubes with 500 μl of 50% ethanol to dislodge the sporangia (Cohen, Meron, Mor, & Zuriel, 2003). Sporangial count was then determined using a hemocytometer, and sporangia production was expressed as spores per cm^2^ leaf area. A spore concentration of about 5 × 10^3^ spores/cm^2^ and a disease severity rating of ≥50% were considered as compatible (+) reaction, and an absence of sporulation or very low sporulation (~3 × 10^3^ spores/ml) and <20% disease severity were considered as incompatible (−) reaction. Pathotype designation was based on the pattern of compatible and incompatible reactions of each isolate with the differential host set (Cohen et al., 2003, 2015; Thomas et al., 1987).

### 2.4 DNA extraction and sequencing

Seven days after inoculation, leaves inoculated with individual reference isolates were rinsed with sterile distilled water using a Preval sprayer (Complete Unit 267; Precision Valve Corporation, Yonkers, NY). The sporangial suspension was then filtered using a sterile mira cloth to remove any plant material, centrifuged, and subjected to DNA isolation using Qiagen DNeasy Plant Mini Kit (Qiagen Corporation, Maryland, USA). Total genomic DNA was quantified using Qubit 2.0 fluorometer (Life Technologies, Grand Island, NY). DNA quality and integrity were further checked by PCR amplification and sequencing of β‐tubulin, which harbors species‐specific variation (Quesada‐Ocampo et al., 2012). The sequenced region was searched against the NCBI nonredundant database using BLASTn, and the identity of each isolate was confirmed as *P. cubensis*. At least 1 μg of DNA from each sample was submitted to the Genomic Sciences Laboratory at North Carolina State University for sequencing, using 150‐bp paired‐end sequencing on the Illumina HiSeq platform. Samples for all isolates were barcoded and multiplexed with nine samples per lane. The watermelon isolate, 2013E1, was resequenced using 300‐bp paired‐end MiSeq due to low read coverage in the first run. DNA extraction, library preparation, and genome sequencing of the *P. humuli* isolate were performed as previously described (Withers et al., 2016). The *P. humuli* isolate used in this study was originally isolated from hop in Oregon (Table 1).

### 2.5 Comparative whole‐genome sequence analysis

A publicly available draft genome assembly of *P. cubensis*, MSU‐1 (67.9 Mbp) isolate originally isolated from cucumber, was used for reference‐guided comparative genome analyses (Savory et al., 2012). A complete mitochondrial genome assembly (38,553 bp) of *P. cubensis* (Lu, Hu, & Wang, 2015) was used as the reference for aligning mitochondrial reads generated from the reference samples.

**Figure 1:**
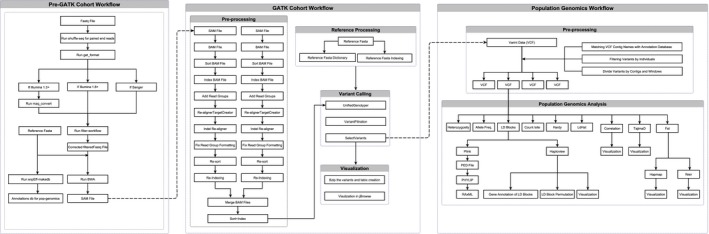
A schematic description of the pipeline developed in Mobyle SNAP workbench used for read processing, reference‐guided alignment, variant calling, and population genomics of *Pseudoperonospora cubensis* isolates. BAM: Binary Alignment/Map; BWA: Burrows–Wheeler Aligner; *F*_ST_: measure of genetic differentiation among populations; Hardy: Hardy–Weinberg test; LD: linkage disequilibrium; SAM: Sequence Alignment/Map; VCF: Variant Call Format; PED: Pedigree; Weir: *F*_ST_ estimate (Weir & Cockerham, 1984)

A pipeline in Mobyle SNAP Workbench that was developed (Aylor, Price, & Carbone, 2006; Monacell & Carbone, 2014; Price & Carbone, 2005) previously for read filtering and variant discovery (Figure 1) was used in this study. Raw sequence reads were error‐corrected using Quake (Kelley, Schatz, & Salzberg, 2010) and filtered based on a Phred‐quality score >20 using the FASTX‐Toolkit (Gordon & Hannon, 2010). Filtered read pairs were interleaved into a single FASTQ file using the shuffleSequences.pl script in Velvet version 1.2.10 (Zerbino & Birney, 2008). Corrected and filtered reads were aligned to the MSU‐1 reference genome (Savory et al., 2012) and the reference mitochondrial genome (Lu et al., 2015) using the Burrows–Wheeler Aligner (Li & Durbin, 2009). Sequence Alignment/Map (SAM) files were assembled into cohorts and genotyped using the UnifiedGenotyper variant discovery pipeline in GATK v2.4‐9 (DePristo et al., 2011). The Variant Call Format (VCF) file output from GATK was then used to conduct population genomic analyses using PLINK (Purcell et al., 2007), VCFTOOLS (DePristo et al., 2011), and HAPLOVIEW (Barrett, Fry, Maller, & Daly, 2005), and to conduct phylogenetic analyses using RAxML (Stamatakis, 2006). Population genomic analyses were based on the ten longest contigs ranging from 59 to 106 kbp in size. Estimates of Tajima's D statistic, allele frequency, linkage disequilibrium (LD), heterozygosity, and *F*_ST_ were based on a sliding window analysis across the contigs. An ancestral recombination graph (ARG) was reconstructed using the branch and bound method implemented in Beagle (Lyngsø, Song, & Hein, 2005) and was based on a window of 100 single nucleotide polymorphisms (SNPs) from a contiguous stretch of DNA on each contig. If there were too many recombination events for branch and bound to go to completion, a heuristic implementation of the Beagle algorithm, KWARG (Lyngsø et al., 2005), was used. Ancestral recombination graphs capture both mutation and recombination in the ancestral history of the sample and provide insights into the putative parents of recombinant haplotypes (Carbone, Jakobek, Ramirez‐Prado, & Horn, 2007; Moore, Singh, Horn, & Carbone, 2009). Although our study sample includes only nine isolates of *P. cubensis*, much of the molecular sequence variation examined spans the population‐species interface. Computer simulation studies (Felsenstein, 2006; Pluzhnikov & Donnelly, 1996) have shown that even a small sample size of eight isolates yields accurate estimates of population parameters and processes, such as the neutral mutation rate and recombination, provided that more sites and more sequences are examined when recombination is present. In predominantly clonal organisms, LD is widespread and this necessitates examining at least three or more widely separated loci on chromosomes or scaffolds (more if LD blocks are large) for accurately reconstructing the history of recombination. With recent population divergence, there is insufficient variation at a single locus and as our sample size is limiting, we examined all available segregating sites found in the ten longest genome scaffolds for reconstructing the underlying phylogeny and for estimating population parameters.

### 2.6 Coalescent analysis

The ancestral history of *P. cubensis* and *P. humuli* was inferred using Genetree (Griffiths & Tavaré, 1994) implemented in Mobyle SNAP workbench. Coalescent analysis was used to infer rooted gene genealogies showing the relative ages of mutations, haplotypes, and clades. Genealogies for segments of the mitochondrial and nuclear genomes were inferred separately. Specifically, nuclear gene genealogies were inferred for each of the ten longest contigs using windows of ~100 SNPs from the start, middle region, and end of each contig. All SNPs from the mitochondrial genome (~38 kbp) were used for genealogical analysis. Compatibility among pairs of SNPs was used to determine the largest nonrecombining partition of compatible SNPs for each contig using the CladeEx program (Bowden, Price, & Carbone, 2008). The population mean mutation rate was based on Watterson's θ estimator calculated for each analysis window (Griffiths & Tavaré, 1994). The relative probabilities of all rooted genealogies were calculated by performing 1 × 10^6^ simulations of the coalescent assuming panmixis and constant population size that was tested a priori using the Tajima's D statistic. Three independent coalescent runs with different starting random number seeds were performed to select the best‐rooted gene genealogies for inference of ancestral relationships.

## 3 RESULTS

### 3.1 Mating type determination

Of the nine reference isolates tested, four isolates produced oospores when they were crossed with a known A2 mating type tester isolate and were assigned to the A1 mating type. The remaining five isolates produced oospores only when they were paired with the A1 tester isolate and were designated as the A2 mating type. Two isolates collected from cucumber (A11 and 08A1) and one isolate from cantaloupe (2013B17) and pumpkin (2013C3) belonged to A1 mating type. Two isolates from butternut squash (D3 and 2013D6), two from acorn squash (08F1 and 2013F2), and one isolate collected from watermelon (2013E1) were found to belong to the A2 mating type (Table 1).

### 3.2 Pathotype determination and designation

All isolates collected from cucumber, cantaloupe, and giant pumpkin were compatible with *Cucumis* spp., *C. maxima,* and *L. siceraria* but incompatible with *C. pepo, C. moschata, C. lanatus,* or any other host types. Isolate A11 collected from cucumber and 2013F2 collected from acorn squash were found to be compatible with *B. hispida*. Isolate 2013E1 collected from watermelon was compatible with *Cucumis* spp., *C. lanatus,* and *C. maxima* but incompatible with any other host types tested, while all isolates collected from squashes were found to be compatible with all *Cucurbita* spp., *Cucumis* spp., and *C. lanatus*. Based on the pattern of compatibility and incompatibility with the host differential set, 2013B17 and 2013C3 were designated as pathotype 1, while A11 and 08A1 were designated as pathotype 3 (Table 1). Isolates 2013E1 and 08F1 were classified as pathotypes 4 and 6, respectively, while the remaining isolates were designated as pathotype 5 (Table 1).

### 3.3 Population genomics

**Figure 2:**
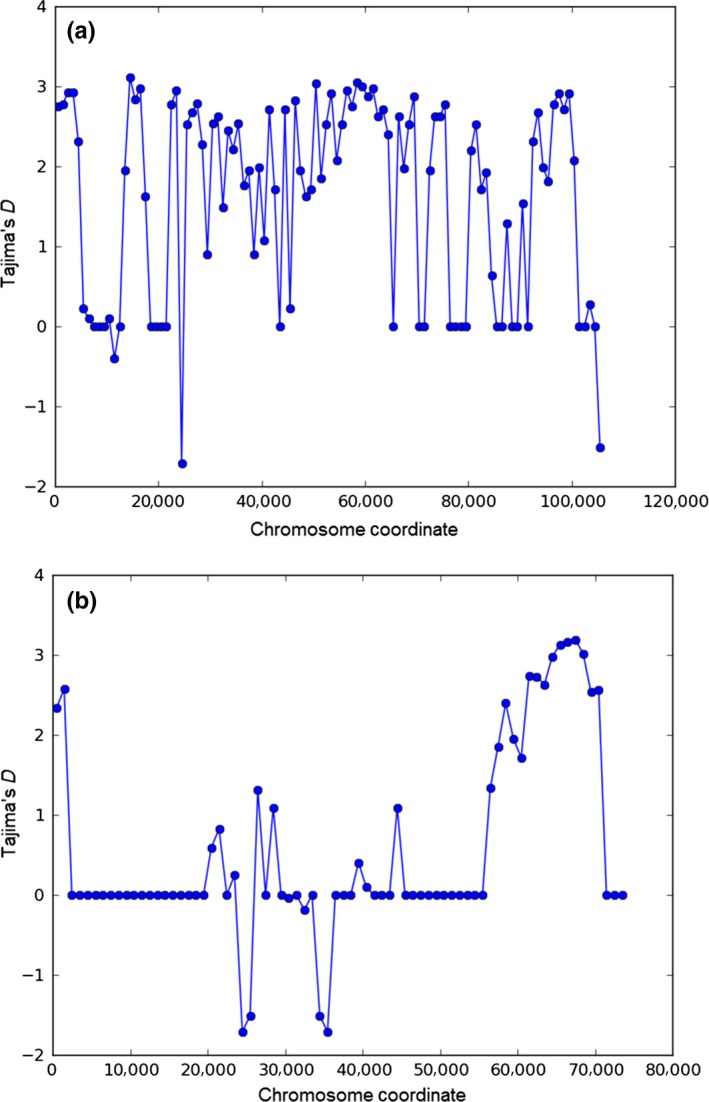
Average values of Tajima's D statistic estimated for every 1 kbp across contigs; a) contig 1 of size 106.0 kbp with 680 SNPs; b) contig 3 of size 75.3 kbp with 256 SNPs

**Figure 3:**
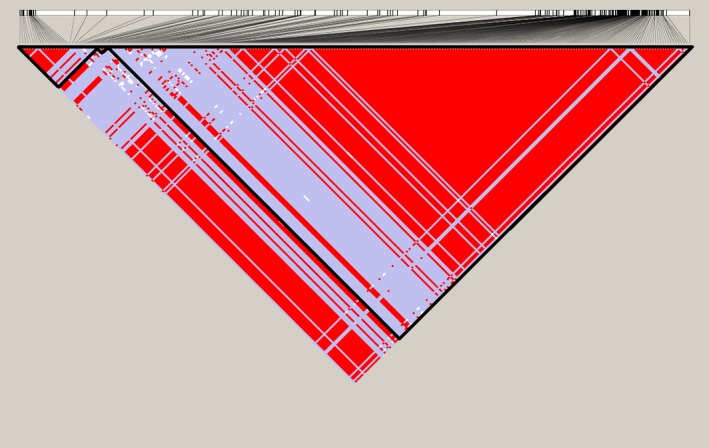
Linkage disequilibrium (LD) plot for contig 3 (73.4 kbp; 256 SNPs) generated using the solid spine algorithm, showing at least two distinct LD blocks. The red color indicates a coefficient of determination, *R* ^2 ^= 1, while the gray color indicates *R* ^2 ^< 1, among sites

Population genomic analyses were based on 3,356 SNPs spanning the ten largest contigs across the nine sequenced genomes. Binary Alignment/Map (BAM) files for alignments of each of the ten sequenced genomes to the MSU‐1 reference genome have been submitted to the NCBI sequence read archive (Accession Nos. SAMN06210292, SAMN06210293, SAMN06210294, SAMN06210295, SAMN06210296, SAMN06210297, SAMN06210298, SAMN06210299, SAMN06210300, SAMN06210301). Population parameter estimates were based on a 1‐kbp sliding window analysis. Tajima's D statistic was zero for windows spanning many genomic regions, which is consistent with neutrality. However, there was a signature of population size expansion or positive selection (Tajima's *D* > 0) (Figure 2) in some regions that were identified as a hypothetical protein or protein kinase. Plots of coefficients of determination (*R* ^2^) revealed distinct LD blocks in seven of the 10 contigs, which suggests recent genetic exchange and recombination in *P. cubensis* (Figure 3). Approximately 37% of the SNPs were heterozygous across both lineages, based on evidence from paired‐end reads. In addition, 93% of the SNPs were heterozygous among lineage II isolates.

**Figure 4:**
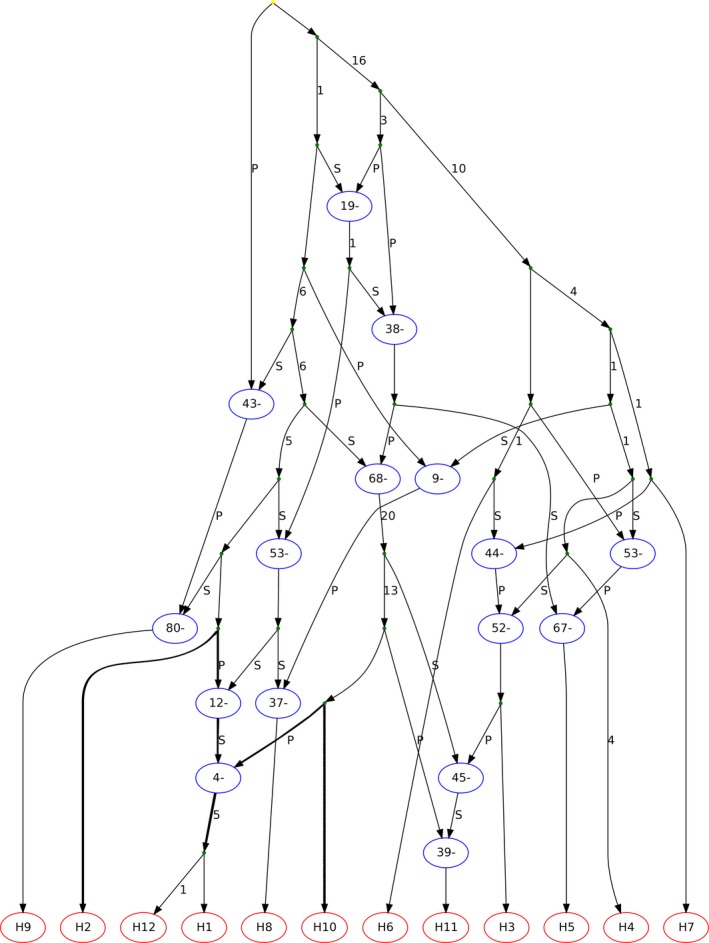
Ancestral recombination graph (ARG) inferred using the Kwarg heuristic search method for 100 contiguous SNPs spanning the middle segment of nuclear contig 7. The ARG shows one possible reconstruction of mutation and recombination paths giving rise to the sampled haplotypes. The direction of paths is from the top (past) to the bottom (present). The paths leading to the recombination nodes (ovals) are labeled with a P (prefix) or S (suffix), indicating the 5__ and 3__ segments of the recombinant sequence, respectively. The numbers displayed along the paths indicate the number of mutation events. The number shown inside the label refers to the variable position immediately to the left of the recombination breakpoint. The ARG shows a putative hybrid origin of *P. cubensis* lineage II (haplotypes H1 and H12) with haplotype H2, comprising isolates belonging to *P. cubensis* lineage I and haplotype H10 (*P. humuli*) as putative parents. The paths leading to putative parents are shown in bold, and they represent the minimal number of mutations and recombinant events from parents to recombinant. Haplotype identity is as follows: H1: 08A1‐1, 2013B17‐1, 2013B17‐2, 2013C3‐1, A11‐1, H2: 08F1‐1, 2013D6‐1, 2013F2‐1: H3 08F1‐2, H4: 2013E1‐2, H5: 2013D6‐2, H6: D3‐2, H7: 2013F2‐2, H8: 2013E1‐1, H9: D3‐1, H10: Ph‐1, H11: Ph‐2, and H12: 08A1‐2, 2013C3‐2, A11‐2

An ancestral recombination graph (ARG) indicated extensive recombination among inferred single‐locus haplotypes of *P. cubensis* and *P. humuli*. There was evidence of recombination among lineage I haplotypes of *P. cubensis*. However, mutation alone separated haplotypes comprising lineage II, as was observed for 27 of 29 regions examined. Four of the ten contigs provided evidence for a hybrid origin of lineage II. For example, the ARG inferred for a DNA segment on contig 7 indicates that one or more crossovers involving haplotypes H2, H8, and H10 followed by mutation gave rise to the clade that comprises haplotypes H12 and H1 (Figure 4). One putative parental haplotype H2 belongs to *P. cubensis* lineage I, while the other putative parental haplotype H10 represents *P. humuli* and the recombinant haplotypes H12 and H1 comprise *P. cubensis* lineage II isolates. An alternative, but less parsimonious parental haplotype, is H8 from *P. cubensis* lineage I, as the path from haplotype H8 to the hybrid haplotypes H12 and H1 requires three recombination events instead of one from *P. humuli* haplotype H10 (Figure 4).

### 3.4 Phylogenetic analysis

**Figure 5:**
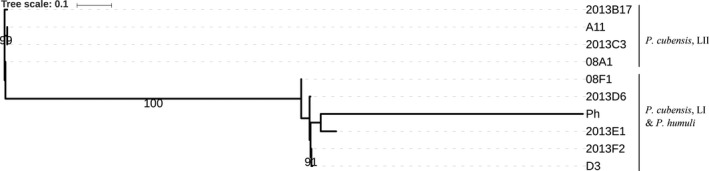
Best maximum likelihood phylogenetic tree based on variation in the mitochondrial genome (38.5 kbp, total size; 392 SNPs) for nine isolates of *Pseudoperonospora cubensis* and one isolate of *P. humuli*. Isolates of *P. cubensis* belonging to lineages I and II and an isolate of *P. humuli* are indicated on the tree, with the tree scale indicating the base substitutions per site

**Figure 6:**
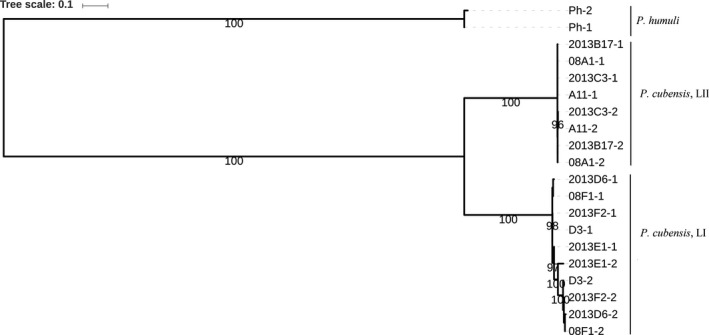
Best maximum likelihood phylogenetic tree based on the nuclear allelic variation in contig 1 (106 kbp; 680 SNPs) for nine isolates of *Pseudoperonospora cubensis* rooted with *P. humuli*. Isolates of *P. cubensis* belonging to lineage I and lineage II are shown on the tree for which the tree scale indicates the base substitutions per site

The mitochondrial phylogeny based on 392 SNPs showed that lineage I of *P. cubensis* shared a recent common ancestor with *P. humuli* (Figure 5). Nuclear phylogenetic analyses based on 3,356 SNPs provided evidence of two distinct evolutionary lineages of *P. cubensis* that are associated with specific hosts, mating types, and pathotypes (Figure 6). Lineage II isolates were associated with cucumber, cantaloupe, and pumpkin, while lineage I isolates were associated with squash and watermelon. The lineage of *P. cubensis* was also found to be associated with mating types and pathotypes. All isolates in lineage II belonged to the A1 mating type and pathotypes 1 or 3, while all isolates in lineage I belonged to the A2 mating type and pathotype 4 or 5. Allelic variation was low within lineage II isolates (π = 0.005) but high within lineage I isolates (π = 0.059).

### 3.5 Coalescent analysis

**Figure 7:**
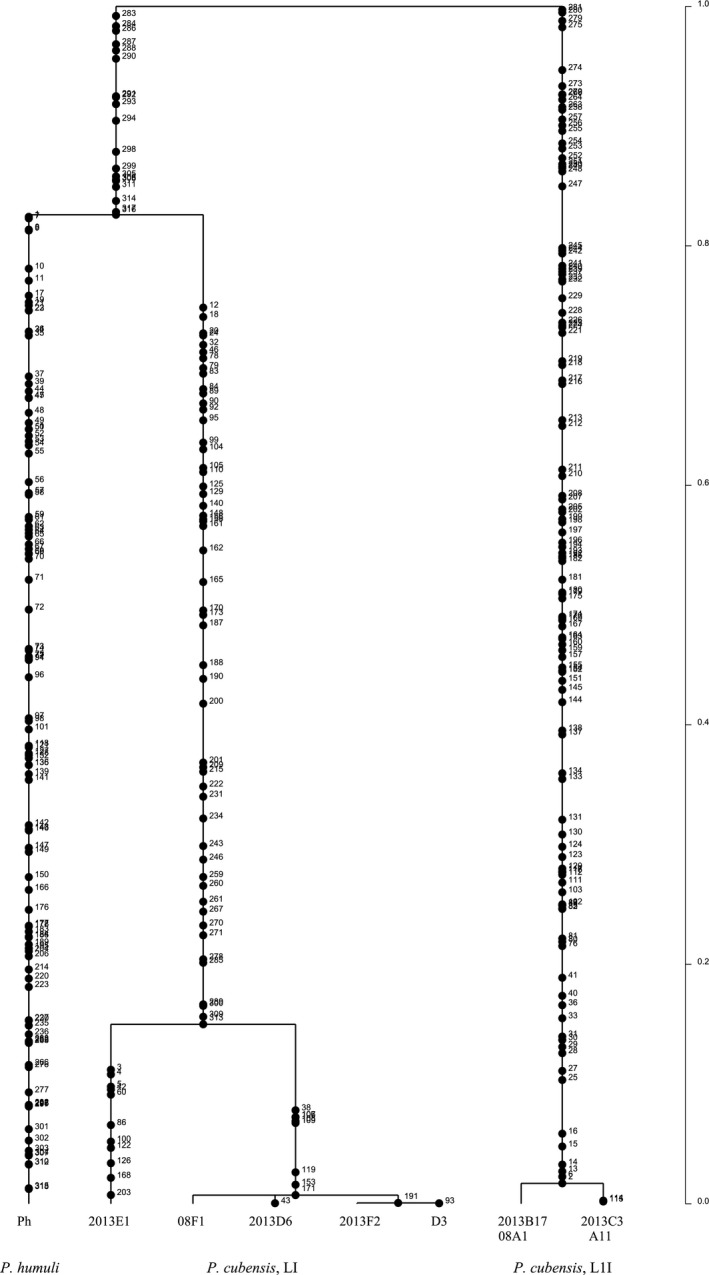
Rooted mitochondrial gene genealogy showing the distributions of mutations of *Pseudoperonospora cubensis* and *P. humuli* in the entire mitochondrial genome (38 kb, 336 SNPs). The numbers indicated along the branches indicate the number of mutation events. The analysis is based on the highest probability tree from three independent runs using 1 × 10^6^ coalescent simulations. There are three distinct evolutionary lineages: *P. cubensis* lineage I (LI), *P. cubensis* lineage II (LII), and *P. humuli*. Lineage I of *P. cubensis* and *P. humuli* shared a common ancestor in the past. The timescale is in coalescent units, and the direction of divergence is from top (past) to the bottom (present)

**Figure 8:**
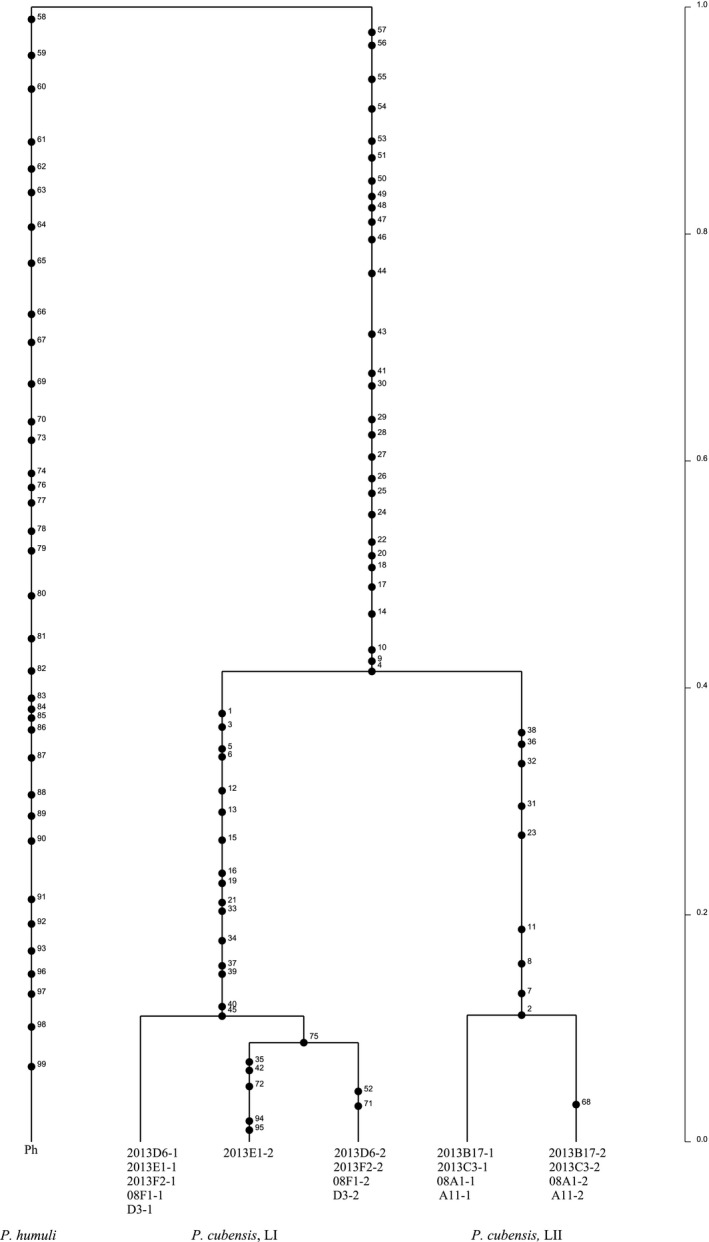
Rooted nuclear genealogy depicting the distribution of mutations of *Pseudoperonospora cubensis* and *P. humuli* using 100 contiguous SNPs from one end of contig 1, with mutations numbered along the branches. The analysis was performed using 1 × 10^6^ coalescent simulations, and the best tree was based on three independent runs. Lineage I and lineage II of *P. cubensis* have diverged from a common ancestor, which coincides with the speciation event separating *P. humuli* and *P. cubensis*. The timescale is in coalescent units, and the direction of divergence is from top (past) to the bottom (present)

**Figure 9:**
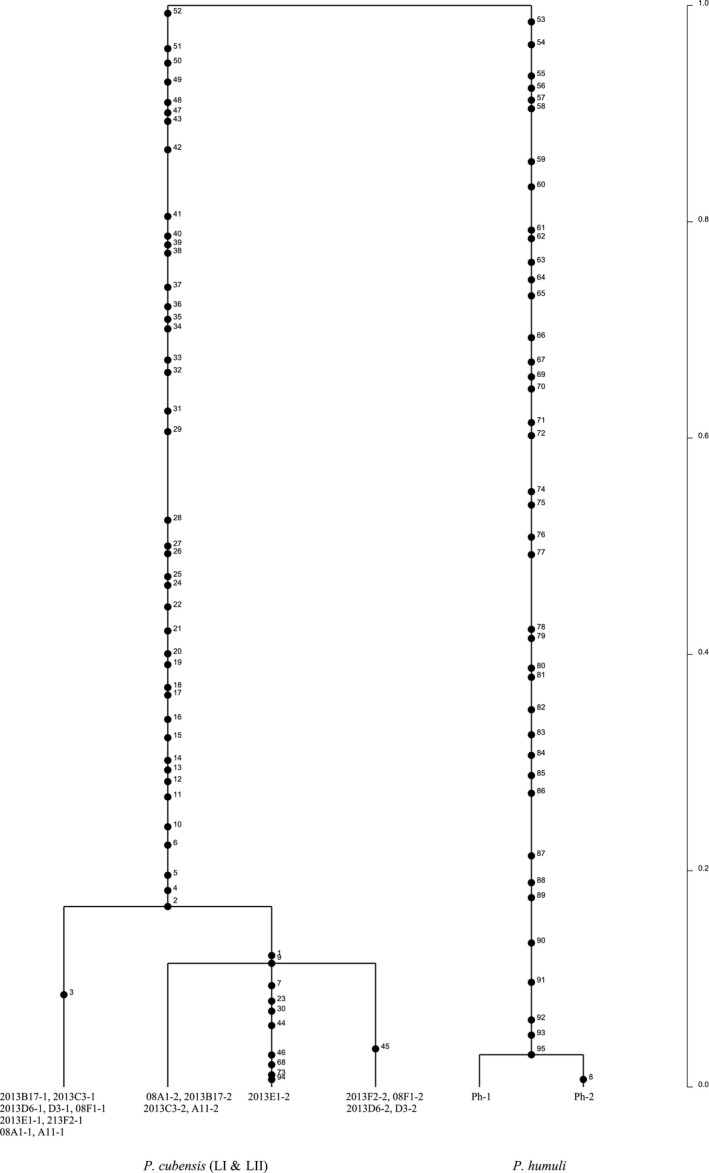
Rooted nuclear gene genealogy showing the distribution of mutations of *Pseudoperonospora cubensis* and *P. humuli* using 100 contiguous SNPs from one end of contig 4. Mutations are numbered along the branches. The analysis is based on 1 × 10^6^ coalescent simulations, and the best tree was generated based on three independent runs. The genealogy clearly separates the speciation event between *P. humuli* and *P. cubensis* (many fixed polymorphisms) in the distant past (top) from population divergence events (few polymorphisms) within each species on a more recent timescale (bottom). Alleles of lineage I and lineage II of *P. cubensis* are shared between isolates, and the distinction between the lineages is not apparent at this segment of the nuclear genome

**Figure 10:**
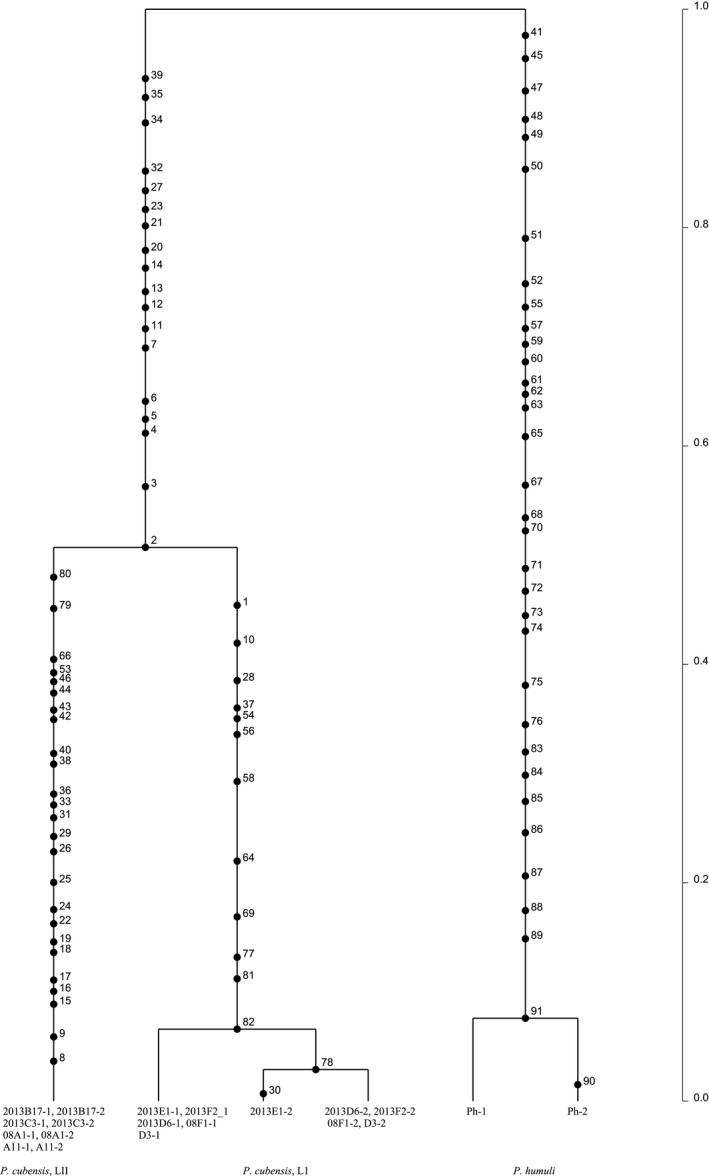
Rooted nuclear genealogy showing the distribution of mutations of *Pseudoperonospora cubensis* and *P. humuli* using 100 contiguous SNPs from the first part of contig 9. The analysis is based on 1 × 10^6^ coalescent simulations, and the best tree was generated based on three independent runs. Lineage I and lineage II of *P. cubensis* diverged from a recent common ancestor, which coincides with the speciation event separating *P. humuli* from *P. cubensis*. The timescale is in coalescent units, and the direction of divergence is from top (past) to the bottom (present)

**Figure 11:**
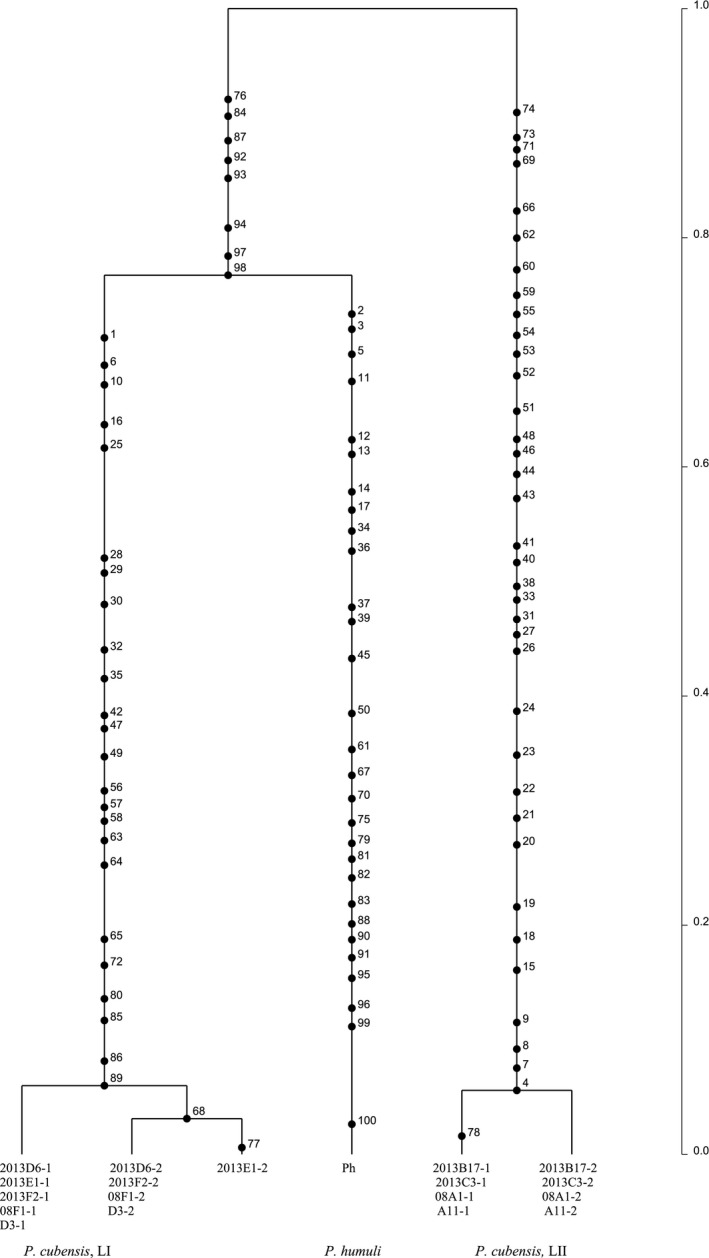
Rooted nuclear gene genealogy showing the distribution of mutations of *Pseudoperonospora cubensis* and *P. humuli* based on 100 contiguous SNPs from one end of contig 3, with mutations numbered along the branches. The analysis is based on the highest probability tree from three independent runs for 1 × 10^6^ coalescent simulations. There are three distinct evolutionary lineages, and lineage I of *P. cubensis* and *P. humuli* shared a common ancestor in the past, which in turn shared a common ancestor with *P. cubensis* lineage II. The timescale is in coalescent units, and the direction of divergence is from top (past) to the bottom (present)

Mitochondrial gene genealogies were based on 336 SNPs and eleven haplotypes that spanned the largest nonrecombining block in the mitochondrial genome of *P. cubensis* and *P. humuli*. Coalescent analysis revealed three distinct evolutionary lineages: *P. cubensis* lineage I, *P. cubensis* lineage II, and *P. humuli* (Figure 7). Although the three lineages are separated by many fixed polymorphisms, lineage I of *P. cubensis* shared a more recent common ancestor with *P. humuli* rather than with its conspecific lineage II. A total of thirty gene genealogies were inferred for the ten longest nuclear contigs. All six gene genealogies inferred for contigs 1 and 2 showed that lineages I and II of *P. cubensis* shared a more recent common ancestor that was distinct from *P. humuli* (Figure 8). This topology was supported in the majority (20 of 30) of nuclear gene genealogies. In some regions that spanned contigs 3, 4, 6, 9, and 10, mutation rates were low (π = 0.04) and *P. cubensis* lineages were indiscernible (Figure 9), while in other regions, mutation rates were higher (π = 0.27) and there was evidence of recombination (or shared haplotypes) between the two lineages of *P. cubensis* (Figure 10). Concordance between the nuclear and mitochondrial gene genealogies was observed for two regions on contigs 3 and 8 (Figure 11).

## 4 DISCUSSION

Genomewide variation in *P. cubensis* has provided evidence for the existence of two distinct evolutionary lineages specialized on different cucurbit host types. Lineage I was associated with isolates infecting squash and watermelon, while lineage II was associated mainly with isolates infecting *Cucumis* spp. Pairing assays of the reference isolates with mating type tester strains showed that all lineage I isolates belonged to mating type A2, while all lineage II isolates belonged to mating type A1. In addition, lineage II was comprised of isolates belonging to pathotypes 1 and 3, whereas lineage I comprised isolates belonging to pathotypes 4, 5, and 6. Although evolutionarily distinct, lineages I and II show a history of genetic exchange and recombination with a closely related sibling species, *P. humuli*. Specifically, there was evidence of recombination between lineage I of *P. cubensis* and *P. humuli* that was supported by a shared common ancestor in the mitochondrial gene genealogy and recombinant hybrids in the inferred nuclear ARGs. This knowledge coupled with the apparent specialization of lineages with host and mating type has implications in the epidemiology and management of CDM.

**Figure 12:**
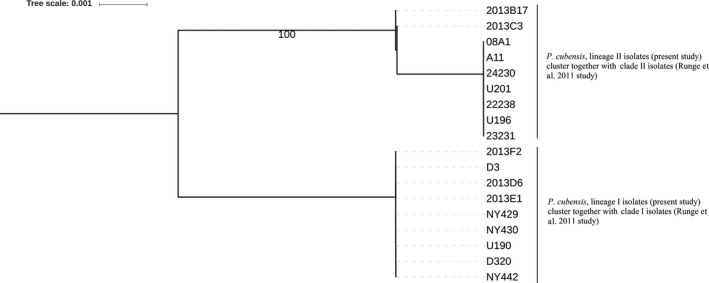
Best maximum likelihood phylogenetic tree based on variation in the *cox*2 region of *Pseudoperonospora cubensis*. Lineage I and lineage II of *P. cubensis* are shown on the phylogenetic tree. Sequence data for the *cox*2 region of isolates labeled as 24230, U201, 22238, U196, 23231, NY429, NY430, U190, D320, and NY442 (from Runge et al., 2011) were obtained from the NCBI database

Previous studies using multilocus phylogenetic analyses of variation in *cox2*,* ypt1,* and nrITS in several similar strains also provided evidence of two clades within *P. cubensis* (Kitner et al., 2015; Runge et al., 2011). Evolutionary lineages I and II identified in the current study were found to be congruent with clades 1 and 2 (Runge et al., 2011), respectively, based on a phylogenetic analysis of sequence data obtained from NCBI (Figure 12). In addition, Kitner et al. (2015) in the Czech Republic observed that all isolates sampled before 2009 were associated with *Cucumis* species, while a broader host range was associated with a subset of isolates collected after 2009. A recent study on the genetic variation between *P. cubensis* and *P. humuli* in the United States used principal component analysis and separated *P. cubensis* isolates into two groups, with one group being composed of isolates from *Cucumis* spp., and the other group comprising isolates from *Cucurbita* spp. (Summers et al., 2015). In the present study, lineage I was composed of isolates from *Cucurbita* spp. and *Citrullus* spp., while lineage II was also composed primarily of isolates from *Cucumis* spp. These results are also similar to those reported by Lebeda et al. (2014), where one clade was composed of isolates primarily from *Cucumis* spp., and the other clade was comprised of isolates from a broader host range. The mechanism controlling host preference in *P. cubensis* is still unknown, and understanding how these lineages are associated with different host types may provide insights into the potential mechanism(s) that control host preference within the *P. cubensis*‐cucurbit pathosystem.

In the present study, phylogenetic analysis showed low allelic variation among lineage II isolates compared to lineage I isolates. The lower genetic diversity in lineage II, comprising of four isolates collected from different hosts across a wide geographic area, is indicative of a recently introduced population, a bottleneck or a selective sweep within *P. cubensis*. Lineage I, comprising of five isolates belonging to pathotypes 4, 5, and 6, showed a high level of genetic diversity. Isolates belonging to pathotypes 4 and 5 had previously been in the United States (Thomas et al., 1987) prior to the resurgence of CDM in 2004. In the United States, CDM were usually observed on squash, pumpkin, and watermelon prior 2004 (Holmes et al., 2006), while disease on cucumber were very minimal prior to 2004 and required very limited fungicide sprays for effective disease control. The latter was largely due to the *dm‐1* resistance gene that had been widely deployed in cucumber since 1960s (Criswell et al., 2010). In present study, only isolates within lineage II were highly compatible with Poinsett 76, a cucumber cultivar that has the *dm‐1* gene. Isolates specialized on *Cucumis* spp. also belonged to pathotypes 1 and 3 that were previously known to exist only in Asia (Thomas et al., 1987). The low allelic variation coupled with the ability to infect cucumber with the *dm‐1* gene suggests that isolates within lineage II may have been responsible for the resurgence of CDM in the United States.

A resurgence of CDM in Europe on cucumber was observed during the second half of 1980s, and pathotypes compatible with *Cucurbita* spp. were later reported in Europe from 2002 (Cappelli, Buonaurio, & Stravato, 2003; Lebeda & Gadasová (2002)). Resurgence of CDM was postulated to have been mediated through anthropogenic means that may have led to the migration of lineage II of *P. cubensis* from East Asia to Europe and from Europe to the United States (Runge et al., 2011). It is plausible that the migration of lineage II from East Asia to Europe and the United States was potentially mediated by the exchange of seed materials as part of a global effort to manage CDM through resistance breeding when it first appeared in Europe (Shetty & Wehner, 2002). A recent study revealed that *P. cubensis* could also be transmitted through seed (Cohen et al., 2014). Thus, it is possible that seeds harboring the pathogen may have inadvertently introduced lineage II isolates from East Asia to Europe and the United States. A more detailed population genetic study employing robust SNP markers and a diverse set of isolates sampled from different cucurbit host types around the world is needed to provide a better understanding of the population structure and migratory pathways of *P. cubensis* on a global scale.

Previous studies (Cohen & Rubin, 2012) have suggested an association between cucurbit host types and mating types of *P. cubensis*. There have also been reports of both mating types being able to infect the same host species suggesting that mating type and virulence might be genetically unlinked (Cohen et al., 2015). In the present study, all lineage II isolates were of the A1 mating type, while all lineage I isolates were of the A2 mating type. However, the association of lineage and cucurbit host type was not very specific. Indeed, both lineage I and lineage II isolates were able to cause infection on susceptible varieties of cucumber, cantaloupe, and giant pumpkin. This suggests that the association between host types and mating types may be a reflection of the differences in the lineages of *P. cubensis* rather than differences mediated through host types. In the United States, the distinctiveness between lineages of *P. cubensis* is more evident, and this may be due to a reproductive isolation due to the absence of lineage II prior to the resurgence of CDM in 2004. Both mating types of *P. cubensis* were reported recently in the United States, and a distinct association between mating type and cucurbit host types was documented (Thomas, Carbone, Cohen, et al., 2017). The distinctiveness between lineages of *P. cubensis* may diminish if recombination becomes more widespread, which could result in the formation of hypervirulent strains with a wider host range (Runge et al., 2011). Although both mating types have now been reported in the United States, the extent of sexual recombination among *P. cubensis* field populations is unknown. Coalescent analysis based on SNP variation in the nuclear genome indicated genetic mixing between lineages I and II of *P. cubensis*. This was also evident from LD plots generated for the ten longest contigs. Distinct LD blocks indicative of genetic recombination were present in seven of the ten contigs examined. Gene genealogies inferred for separate contigs were incongruent, which further supports a history of genetic exchange. Recently, Kitner et al. (2015) reported that over 60% of *P. cubensis* isolates collected in the Czech Republic after 2009 had two heterozygous positions in the nrITS region irrespective of the host of origin, which they postulated as evidence for sexual recombination.

Coalescent‐based analysis of SNPs from mitochondrial genomes revealed a close affinity between *P. humuli* and lineage I of *P. cubensis*. With the exception of a few nuclear regions, which supported this grouping, the majority of nuclear gene genealogies examined supported the monophyly of the two species. One possibility is that *P. humuli* and lineage I of *P. cubensis* initially diverged from a common ancestor (nuclear gene genealogies) and thereafter evolved host specificity on a more recent timescale (mitochondrial gene genealogies), while retaining the ancestral signature of speciation in some genomic regions. Ancestral recombination graph reconstruction indicated a hybrid origin of *P. cubensis* lineage II isolates with lineage I of *P. cubensis* and *P. humuli* as putative parents. However, only a single isolate of *P. humuli* was examined in this study, and several isolates of *P. humuli* are needed to draw robust conclusions. In addition, the potential for interspecific hybridization between two species has not yet been investigated. It will be interesting to sample *P. humuli* and *P. cubensis* isolates where both species coexist and examine whether there is evidence of ongoing genetic exchange between the species, as has been hypothesized in other studies (Choi et al., 2005; Kitner et al., 2015; Runge & Thines, 2012). Previous studies have identified two wild cucurbit relatives, *Bryonia dioica* and *Sicyos angulatus,* as hosts compatible with both *P. humuli* and *P. cubensis* (Runge & Thines, 2012). Susceptible varieties of hop in the United States can also be infected by *P. cubensis* (Mitchell et al., 2011). There could be more undocumented host types that are compatible with the two *Pseudoperonospora* species, and a co‐infection of such hosts may facilitate genetic exchange between the two species. Given the findings from the current study, it is possible that hybridization may have initiated and accelerated the process of host specialization in *P. cubensis*.

## CONFLICT OF INTEREST

The authors declare that they have no conflict of interest.

## DATA AVAILABILITY

Binary Alignment/Map files for alignments of each of the nine sequenced genomes to the MSU‐1 reference genome have been submitted to the NCBI sequence read archive (Accession Nos. SAMN06210292, SAMN06210293, SAMN06210294, SAMN06210295, SAMN06210296, SAMN06210297, SAMN06210298, SAMN06210299, SAMN06210300, SAMN06210301).
